# Dental Organization in Kansas

**Published:** 1871-07

**Authors:** 


### DENTAL ORGANIZATION IN KANSAS.

A State Dental Society was organized May 2nd, 1871, in Lawrence, Kansas, through the instrumentality of Dr. W. H. Marvin, of Topeka, in that State. There were present Dr. J. H. Sawyer, Atchison; Dr. E. C. Fuller, Fort Scott; Dr. A. M. Callahan, Topeka, and Drs. J. B. Wheeler, and J. D. Patterson, of Lawrence. The permanent organization was had as follows: President, Dr. J. B. Wheeler; Vice President, Dr. W. H. Marvin; Recording Secretary, Dr. J. D. Patterson; Corresponding Secretary, Dr. E. C. Fuller; Treasurer, Dr. J. H. Sawyer, under the name and title of the Kansas State Dental Society.

The code of ethics of the American Dental Society was adopted, and a constitution and by-laws were also adopted in harmony with those of standard societies in other States.

Dr. Fuller was elected delegate to the American Dental Association for 1871. A vote of thanks was tendered Dr. Marvin for his efforts in behalf of the organization.

The first semi-annual meeting was set for September 12th, 1871, at Topeka, and the annual meeting at Atchison, May 2nd, 1872.

A general discussion was had on subjects interesting to the profession, and the members were highly gratified with the inaugural movement to place the profession in the State of Kansas on a solid basis, in uniformity with other and older States.

A cordial invitation was extended to dentists throughout the State for co-operation, and the new society adjourned, to meet September 12th.

